# Serum C1q- binding adiponectin in maintenance hemodialysis patients

**DOI:** 10.1186/1471-2369-14-50

**Published:** 2013-02-26

**Authors:** Ken Kishida, Naohiro Kishida, Masaaki Arima, Hideaki Nakatsuji, Hironori Kobayashi, Tohru Funahashi, Iichiro Shimomura

**Affiliations:** 1 Department of Metabolic Medicine, Graduate School of Medicine, Osaka University, 2–2 B-5, Yamada-oka, Suita, Osaka 565-0871, Japan; 2 Kishida Clinic, 5-6-3, Honmachi, Toyonaka, Osaka 560-0021, Japan; 3 Department of Research and Development, Diagnostic Division, Otsuka Pharmaceutical Co., Ltd, Tokushima 771-0195, Japan; 4 Department of Metabolism and Atherosclerosis, Graduate School of Medicine, Osaka University, 2–2 B-5, Yamada-oka, Suita, Osaka 565-0871, Japan

**Keywords:** Adiponectin, C1q, C1q-binding adiponectin, Hemodialysis

## Abstract

**Background:**

Patients on maintenance hemodialysis (HD) have much higher levels of adiponectin (Total-APN). Adiponectin and C1q form a protein complex in human blood, and serum C1q-binding adiponectin (C1q-APN) can be measured. We recently reported that C1q-APN/Total-APN ratio rather than Total-APN correlated with atherosclerosis in diabetics. However, the characteristics of C1q-APN in HD patients remain unclear. The preset study investigated the characteristics of the adiponectin parameters including C1q-APN and also to clarify the relationship between various serum adiponectin parameters and atherosclerotic cardiovascular diseases (ACVD) in HD patients.

**Methods:**

The single cross-sectional study subjects were 117 Japanese patients (males/females = 61/56) on regular HD. Blood Total-APN, high molecular weight-adiponectin (HMW-APN), C1q-APN and C1q concentrations were measured by enzyme-linked immunosorbent assays. ACVD were defined as stroke, coronary and peripheral artery diseases, thoracic and abdominal aneurysms.

**Results:**

Stepwise regression analysis identified high-density lipoprotein-cholesterol (HDL-C) as the only significant and independent determinant of C1q-APN in males, and duration of HD as the only significant and independent determinant of C1q-APN in females. Stepwise regression analysis identified uric acid, low-density lipoprotein-cholesterol and triglyceride as significant and independent determinants of C1q-APN/Total-APN ratio in males, and leukocyte count and HDL-C as significant and independent determinants of C1q-APN/Total-APN ratio in females. Multiple logistic regression analysis identified inorganic phosphorus and C1q-APN or C1q-APN/C1q ratio as significant determinants of ACVD.

**Conclusions:**

Low serum C1q-APN and C1q-APN/C1q ratio, but not C1q-APN/Total-APN ratio, correlated with ACVD in HD patients.

**Trial registration:**

ClinicalTrials.gov: UMIN http://000004318

## Background

Adiponectin is an adipose-specific circulating protein [1], and circulating total-adiponectin levels (Total-APN) are lower in males than in females but not different between pre- and postmenopausal females [2]. Adiponectin has protective properties against diabetes and atherosclerotic cardiovascular disease (ACVD) [3]. ACVD is the major cause of morbidity and mortality in maintenance hemodialysis (HD) patients. Zoccali and our group demonstrated the presence of consistently high levels of Total-APN in HD patients, and that low Total-APN levels were associated with increased risk for ACVD events in HD patients over a follow-up period of 31 months [4]. We recently reported that adiponectin binds with C1q in human blood, and also described the development of a system to measure human serum C1q-binding adiponectin (C1q-APN) [5]. Although circulating Total-APN level is considered a biomarker of the metabolic syndrome [6], serum C1q-APN/Total-APN ratio rather than Total-APN is a novel marker of the metabolic syndrome in male subjects [5]. Serum C1q-APN/Total-APN correlated with polyvascular diseases and coronary artery disease in type 2 diabetics [7,8]. These data suggest that it is important to consider not only the absolute amount of adiponectin but also the levels of relative adiponectin forms in blood. However, the characteristics of C1q-APN, C1q-APN/Total-APN and C1q-APN/C1q in HD patients remain unclear.

The biochemical and haematological parameters should be also important factors in mortality outcomes in HD. The aim of the present study was to determine the relationship between biochemical and haematological parameters, clinical features, and various adiponectin parameters including C1q-APN, and to clarify the relationship between various serum adiponectin parameters and ACVD, in HD patients.

## Methods

### Participants

The study (Victor-J study; #UMIN http://000004318) subjects were 117 Japanese patients (n; males/females = 61/56) on standardized HD for at least 1 year (500–800 mL/min dialysate flow; 250–300 mL/min blood flow; 3.5-4 hours dialysis per session; 3 sessions per week), who visited the Kishida Clinic in February 2010. Patients treated with pioglitazone, which is known to increase serum adiponectin levels [9], and/or patients with clinical evidence of heart failure (defined as dyspnea in addition to two of the following conditions: increased jugular pressure, bi-basal crackles on auscultation, pulmonary venous hypertension, or interstitial edema on the chest x-ray, requiring hospitalization or extra ultrafiltration) were excluded from the study. The Medical Ethics Committee of Osaka University approved the study. Each participant gave a written informed consent.

### Anthropometry and laboratory tests

Anthropometric variables [height and weight] were measured in the standing position and body mass index (BMI) was calculated [=weight (kg) / height (m)^2^]. Waist circumference (WC) at the umbilical level was measured with a non-stretchable tape in late expiration while a standing (in cm). Systolic and diastolic blood pressures (SBP, DBP) were measured with a standard mercury sphygmomanometer on the right or left arm in the supine position after at least 5-minute rest.

Venous blood samples were collected before dialysis session for measurements of serum biochemical parameters, red blood cell counts (RBC), hemoglobin (Hb), hematocrit (Ht), white blood cell counts (WBC), creatinine (Cr), blood urea nitrogen (BUN), albumin (Alb), calcium (Ca), inorganic phosphorus (IP), potassium (K), magnesium (Mg), uric acid (UA), intact parathyroid hormone (intact-PTH), β2-microglobulin (β2MG), blood glucose (BS), total-cholesterol triglyceride (TG), high-density lipoprotein-cholesterol (HDL-C), and C-reactive protein (CRP). Low-density lipoprotein-cholesterol (LDL-C) was calculated using the Friedewald formula. Adjusted-Ca (mg/dL) = measured serum total Ca (mg/dL) – measured serum Alb (mg/dL) + 4.0. Intact-PTH was measured by immunoradiometric assay. For the purpose of the present study, serum samples that were obtained at baseline from each participant were stored promptly at −20°C. After thawing the samples, serum levels of Total-APN and high molecular weight-adiponectin (HMW-APN) were measured by enzyme-linked immunosorbent assay (ELISA) (Human adiponectin ELISA kit, Human HMW-adiponectin ELISA kit, Otsuka Pharmaceutical Co. Tokushima, Japan) [1,10]. C1q-APN and C1q were measured by our handmade ELISA, as reported previously by our group [5]. The intra- and inter-coefficients of variation for C1q-APN ELISA are below 4.6% and 6.7%, respectively [5].

Hypertension (HT) was defined as SBP ≥140 mmHg and/or DBP ≥90 mmHg, or use of antihypertensive medication; (calcium channel antagonist / angiotensin converting enzyme inhibitor or angiotensin receptor blocker / β blockade / diuretics /α blockade =20/11/3/1/5). Diabetes mellitus (DM) was defined as either fasting BS >126 mg/dL, random BS >200 mg/dL, or use of insulin (n = 11) or antidiabetic medication (αGI = 5). Dyslipidemia (DL) represented high LDL-C of >120 mg/dL, hypertriglyceridemia [fasting or postprandial TG of ^3^150 or 200 mg/dL, respectively], and/or low HDL-C of <40 mg/dL.

### Measurements of baPWV, and ABI

Arterial stiffness was assessed by measuring brachial-ankle pulse wave velocity (baPWV) and ankle-brachial index (ABI) using an automatic waveform analyzer (Form/ABI; Omron-Colin Co., Komaki, Japan).

### Definition of ACVD

Documented coronary artery disease (CAD) consisted of one or more of the following criteria: history of stable or unstable angina with documented CAD, history of previous myocardial infarction, percutaneous coronary intervention or coronary artery bypass graft surgery. Documented cerebrovascular disease (CVD) consisted of a hospital or neurologist report with the diagnosis of transient ischemic attack or ischemic stroke. Documented aneurysm of the thoracic aorta (TAA) consisted of aortic diameter >4.5 cm, and documented aneurysm of the abdominal aorta (AAA) represented aortic diameter >4.5 cm, an infra-renal aortic diameter >3.0 cm, or a history of TAA and/or AAA repair. Documented peripheral vascular disease (PAD) represented one or both criteria: current intermittent claudication with an ankle–brachial index of < 0.9 or a history of intermittent claudication together with previous and related intervention, such as angioplasty, stenting, atherectomy, peripheral arterial bypass graft, or other vascular intervention, including amputation. Patients with negative results by either of the above definitions were considered ACVD-free.

### Statistical analysis

Data are presented as mean±SEM. Data of two groups were compared by the Student’s t-test. Differences in frequencies were examined by the χ^2^ test. Relationships between two continuous variables were analyzed using scatter plots and Pearson’s correlation coefficient. The correlations between clinical features and adiponectin parameters were first analyzed by simple regression analysis and then by stepwise regression analysis. The correlations between clinical features and ACVD were first analyzed by simple regression analysis and then by logistic regression analysis. In all cases, *p* values <0.05 and F value >4 were considered statistically significant. All analyses were performed with the JMP Statistical Discovery Software 9.0 (SAS Institute, Cary, NC) or the Statistical Package for Social Sciences (version 11.0.1 J; SPSS, Chicago, IL).

## Results

### Characteristics of male and female HD patients

**Figure 1:**
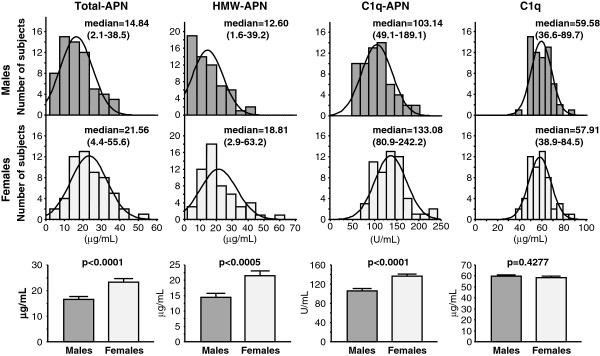
**Comparisons of total adiponectin (Total-APN), high molecular weight-adiponectin (HMW-APN), C1q-binding adiponectin (C1q-APN), and C1q between male and female HD patients.** Data are mean±SEM (range). Data of two groups were compared by the Student’s t-test.

**Figure 2:**
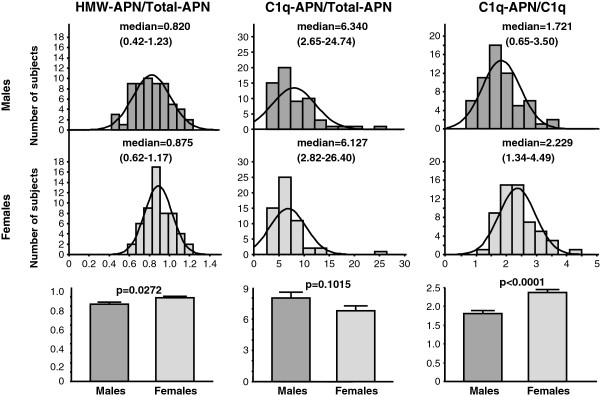
**Comparisons of HMW-APN/Total-APN, C1q-APN/Total-APN, and C1q-APN/C1q between male and female HD patients.** Data are mean±SEM. Data of two groups were compared by the Student’s t-test. Abbreviations as in Figure 1.

Table 1 summarizes the characteristics of male and female HD patients enrolled in this study. The prevalence of ACVD was significantly higher in males than females (57.4% versus 28.6%, p = 0.0299). Serum levels of Total-APN, HMW-APN and C1q-APN were significantly lower in males than in females (16.5 ± 1.1 versus 23.3 ± 1.3 μg/mL, p = 0.0001, 14.5 ± 1.2 versus 21.5 ± 1.5 μg/mL, p = 0.0005, 106.1 ± 4.4 versus 136.3 ± 4.7 units/mL, p < 0.0001, Figure 1). There was no significant difference in serum C1q level between males and females (59.7 ± 1.2 versus 58.3 ± 1.3 μg/mL, p = 0.4277, Figure 1). Serum HMW-APN/Total-APN and C1q-APN/C1q ratios were significantly lower in males than in females (0.82 ± 0.02 versus 0.89 ± 0.02, p = 0.0272, 1.81 ± 0.08 versus 2.37 ± 0.08, p < 0.0001, Figure 2). There was no significant difference in serum C1q-APN/Total-APN ratio between males and females (8.02 ± 0.55 6.81 ± 0.48, p = 0.1015, Figure 2).

**Table 1:** Baseline characteristics of hemodialysis patients

	**Males (n=61)**	**Females (n=56)**	**p value**
Age, years	64±1 (40-81)	66±1 (38-91)	0.2
Body weight (BW), kg	61.5±1.2 (44.5-85.1)	46.7±1.1 (29.0-67.1)	<0.0001
Body mass index (BMI), kg/m2	22.4±0.4 (17.0-31.9)	20.4±0.5 (12.2-27.3)	0
Waist circumference (WC), cm	83.6±0.9 (68.8-100.0)	79.0±1.4 (60.0-104.0)	0.01
Systolic blood pressure (SBP), mmHg	146±3 (83-194)	137±3 (105-180)	0.04
Diastolic blood pressure (DBP), mmHg	75±1 (54-91)	73±1 (54-94)	0.14
Duration of hemodialysis (HD), years	11±1 (1-33)	11±1 (1-35)	0.95
Primary disease (CGN/Diabetic/Others)	n=30/21/10	n=31/10/15	0.64
Hypertension (HT)	n=21	n=11	0.22
Diabetes mellitus (DM)	n=18	n=7	0.12
Dyslipidemia (DL)	n=8	n=8	0.9
Smoking (none-/ex-/current-smoker)	n=12/34/15	n=52/3/1	0.08
ACVD	n=35	n=16	0.03
(CVD/CAD/TAA+AAA/PAD)	(n=14/32/3/18)	(n=6/14/1/12)	
Red blood cell count (RBC) x104, /mL	348±7 (280-532)	338±4 (256-406)	0.19
Hemoglobin (Hb), mg/dL	11.2±0.1 (9.7-14.5)	10.9±0.1 (8.6-11.7)	0.04
Hematocrit (Ht), %	33.6±0.4 (29-45)	33.0±0.2 (26-36)	0.25
White blood cell count (WBC), /mL	5931±267 (3300-10700)	5711±238 (3100-9600)	0.54
Creatinine (Cr), mg/dL	11.8±0.3 (6.3-19.0)	9.7±0.2 (6.3-13.3)	<0.0001
Blood urea nitrogen (BUN), mg/dL	65.8±2.0 (32-104)	67.0±2.0 (37-98)	0.66
Albumin (Alb), g/dL	3.8±0.03 (3.3-4.4)	3.7±0.04 (3.0-4.6)	0.12
Adjusted-calcium (Ca), mg/dL	9.3±0.1 (8.1-11.2)	9.4±0.1 (8.5-10.4)	0.62
Inorganic phosphorus (IP), mg/mL	5.9±0.2 (3.0-9.0)	5.5±0.1 (3.7-7.8)	0.07
Potassium (K), mEq/L	5.1±0.1 (3.6-7.0)	5.2±0.1 (3.7-6.3)	0.76
Magnesium (Mg), mg/dL	2.9±0.1 (2.1-4.1)	2.9±0.1 (2.1-3.8)	0.74
Uric acid (UA), mg/dL	7.0±0.1 (5.3-9.7)	7.2±0.1 (5.0-9.3)	0.35
Intact parathyroid hormone (intact-PTH), pg/mL	114±13 (9.4-412)	139±14 (3.2-389)	0.18
β2-microglobulin (β2MG), mg/L	27.0±0.8 (15.5-38.6)	26.4±0.6 (17.8-38.2)	0.53
Blood glucose (BS), mg/dL	121±5 (68-302)	98±4 (63-239)	0
Low-density lipoprotein-cholesterol (LDL-C), mg/dL	82±3 (32-168)	83±3 (35-147)	0.82
Triglyceride (TG), mg/dL	117±10 (26-400)	104±7 (39-278)	0.28
High-density lipoprotein-cholesterol (HDL-C), mg/dL	48±2 (24-92)	57±2 (19-87)	0
C-reactive protein (CRP), mg/dL	0.54±0.09 (0.30-3.62)	0.41±0.05 (0.30-1.85)	0.24
Ankle-brachial index (ABI)	1.08±0.03 (0.57-1.36)	1.07±0.03 (0.40-1.44)	0.81
baPWV, cm/sec	1970±77 (1166-3835)	2034±121 (4805-5971)	0.66

Data are mean ± SEM (range), or number of subjects.

*ACVD*; atherosclerotic cardiovascular diseases (stroke, coronary and peripheral artery diseases, thoracic and abdominal aneurysms), *CVD*; cerebrovascular disease, *CAD*; coronary artery disease, *TAA*; aneurysm of the thoracic aorta, *AAA*; aneurysm of the abdominal aorta, *PAD*; peripheral vascular disease, *CGN*; chronic glomerulonephritis.

**Figure 3:**
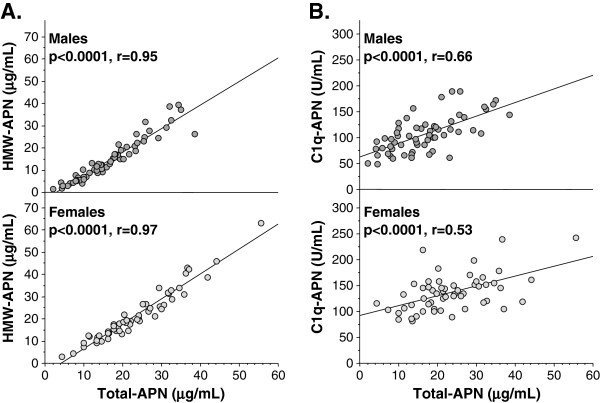
**Correlations between HMW-APN, C1q-APN and Total-APN in male (A) and female (B) HD patients.** Relationships between two continuous variables were analyzed using scatter plots and Pearson’s correlation coefficient. Abbreviations as in Figure 1.

Serum HMW-APN levels correlated significantly and positively with serum Total-APN level both in males and females (Figure 3A). Serum C1q-APN levels correlated significantly and positively but weakly with serum Total-APN level both in males and females (Figure 3B). Serum C1q-APN levels also correlated significantly and positively with serum HMW-APN level both in males and females (r = 0.70, p < 0.0001; r = 0.57, p < 0.0001, respectively, data not shown).

### Correlation between serum adiponectin parameters and clinical features in males

We investigated the correlations between serum adiponectin parameters and clinical features in males (Table 2). Total-APN and HMW-APN correlated significantly and negatively with BMI, WC, WBC, Cr, UA, TG and CRP, and positively with adjusted-Ca and HDL-C. Stepwise regression analysis that included BMI, WC, WBC, Cr, UA, adjusted-Ca, TG, HDL-C, and CRP identified UA, TG and HDL-C as significant and independent determinants of Total-APN, and TG and HDL-C as significant and independent determinants of HMW-APN. C1q-APN correlated significantly and negatively with BMI, WC, UA and TG, and positively with HDL-C. Stepwise regression analysis that included WC, WBC, UA, TG, and HDL-C identified HDL-C as the only significant and independent determinant of C1q-APN. C1q correlated significantly and negatively with age and adjusted-Ca, and positively with BMI, WC, SBP, duration of HD, WBC, IP, β2MG and TG. Stepwise regression analysis that included age, WC, SBP, duration of HD, WBC, adjusted-Ca, IP, β2MG, and TG identified duration of HD, β2MG and TG as significant and independent determinants of C1q.

**Table 2:** Correlations between various adiponectin parameters and clinical features in male patients on maintenance hemodialysis

	**Total-APN**			**HMW-APN**			**C1q-APN**			**C1q**			**HMW-APN/Total-APN**			**C1q-APN/Total-APN**			**C1q-APN/C1q**		
	***Univariate***		***Multivariate***	***Univariate***		***Multivariate***	***Univariate***		***Multivariate***	***Univariate***		***Multivariate***	***Univariate***		***Multivariate***	***Univariate***		***Multivariate***	***Univariate***		***Multivariate***
	**r**	**p**	**F**	**r**	**p**	**F**	**r**	**p**	**F**	**r**	**p**	**F**	**r**	**p**	**F**	**r**	**p**	**F**	**r**	**p**	**F**
Age		0.07			0.11			0.81		-0.39	0	1.7		0.6		-0.36	0	0.15		0.18	
BMI	-0.47	<0.0001	-	-0.4	0	-	-0.27	0.03	-	0.33	0.01	-	-0.28	0.03	-	0.54	<0.0001	-	-0.38	0	-
WC	-0.49	<0.0001	0.97	-0.42	0	0.39	-0.28	0.03	0.49	0.31	0.02	2.6	-0.28	0.03	0.75	0.5	<0.0001	2.31	-0.37	0	2.93
SBP		0.85			0.56			0.26		0.27	0.03	0.02		0.44			0.1			0.69	
Duration of HD		0.2			0.15			0.9		0.31	0.01	11.23		0.3			0.09			0.43	
Hb		0.21			0.14			0.98			0.1			0.82			0.51			0.27	
Smoking (current)		0.29			0.43			0.28			0.62			0.1			0.15			0.43	
WBC	-0.43	0	0	-0.35	0.01	0.04		0.06		0.37	0.01	1.1		0.47		0.36	0.01	3.8	-0.39	0.01	0.7
Cr	-0.45	0	3.96	-0.39	0	1.62		0.28			0.16		-0.28	0.03	2.34	0.51	<0.0001	7.56		0.2	
BUN		0.26			0.16			0.4			0.43			0.05			0.47			0.7	
Alb		0.93			0.96			0.53			0.79			0.94			0.7			0.84	
adjusted-Ca	0.33	0.02	0.84	0.33	0.02	0.65		0.11		-0.3	0.04	0.89		0.11		-0.28	0.05	0.13	0.32	0.03	1.51
IP		0.4			0.68			0.85		0.3	0.04	0.02		0.45		0.32	0.03	0		0.73	
K		0.13			0.07			0.93			0.68		0.31	0.03	2.35		0.12			0.93	
Mg		0.77			0.68			0.69			0.15			0.2			0.13			0.67	
UA	-0.5	0	5.13	-0.44	0	2.46	-0.36	0.01	3.64		0.45			0.37		0.4	0	4.26	-0.34	0.02	1.65
Intact-PTH		0.52			0.26			0.29			0.06			0.14			0.09			0.69	
β2MG		0.09			0.19			0.46		0.43	0	14.13		0.72		0.37	0.01	1.15		0.54	
BS		0.38			0.31			0.59			0.51			0.54			0.95			0.88	
LDL-C		0.06			0.24			1			0.66			0.92		-0.29	0.03	5.24		0.87	
TG	-0.55	<0.0001	10.38	-0.5	<0.0001	9.06	-0.34	0.01	1.65	0.47	0	18.73	-0.51	0.05	0.59	0.65	<0.0001	34.07	-0.45	0	13.46
HDL-C	0.42	0	5.76	0.44	0	8.55	0.32	0.01	8.18		0.08		0.4	0	12.53	-0.42	0	1.77	0.36	0	1.86
CRP	-0.31	0.03	0.04	-0.29	0.04	0.03		0.86			0.1			0.09		0.33	0.02	0.49		0.36	

Parameters with p<0.05 were subsequently entered into multiple regression analysis as significant and independent variables. Parameters with F>4.0 were significant and independent variables. Abbreviations as in Table 1.

We recently showed that serum C1q-APN/Total-APN ratio is associated the metabolic syndrome [5]. We next investigated the correlations between each adiponectin ratio and clinical features in males. The HMW-APN/Total-APN ratio correlated significantly and negatively with BMI, WC, Cr and TG, and positively with K and HDL-C. Stepwise regression analysis that included WC, WBC, Cr, TG, K, and HDL-C identified HDL-C as the only significant and independent determinant of HMW-APN/Total-APN ratio. C1q-APN/Total-APN ratio correlated significantly and negatively with age, adjusted-Ca, LDL-C and HDL-C, and positively with BMI, WC, WBC, Cr, IP, UA, β2MG, TG and CRP. Stepwise regression analysis that included age, WC, WBC, Cr, adjusted-Ca, IP, UA, β2MG, TG, HDL-C, and CRP identified Cr, UA, LDL-C and TG as significant and independent determinants of the C1q-APN/Total-APN ratio. The C1q-APN/C1q ratio correlated significantly and negatively with BMI, WC, WBC, UA and TG, and positively with adjusted-Ca and HDL-C. Stepwise regression analysis that included WC, WBC, adjusted-Ca, UA, TG, and HDL-C identified TG as the only significant and independent determinant of C1q-APN/C1q.

### Correlation between serum adiponectin parameters and clinical features in females

Next, we investigated the correlations between serum adiponectin parameters and clinical features in females (Table 3). Total-APN correlated significantly and negatively with WC, WBC and TG, and positively with IP and HDL-C. HMW-APN correlated significantly and negatively with WC, WBC and TG, and positively with HDL-C. Stepwise regression analysis identified WBC and HDL-C as significant and independent determinants of both Total-APN and HMW-APN. C1q-APN correlated significantly and positively with the duration of HD and IP. Stepwise regression analysis identified the duration of HD as the only significant and independent determinant of C1q-APN. C1q correlated significantly and negatively with UA only.

**Table 3:** Correlations between various adiponectin parameters and clinical features in female patients on maintenance hemodialysis

	**Total-APN**			**HMW-APN**			**C1q-APN**			**C1q**			**HMW-APN/Total-APN**			**C1q-APN/Total-APN**			**C1q-APN/C1q**		
	***Univariate***		***Multivariate***	***Univariate***		***Multivariate***	***Univariate***		***Multivariate***	***Univariate***		***Multivariate***	***Univariate***		***Multivariate***	***Univariate***		***Multivariate***	***Univariate***		***Multivariate***
	**r**	**p**	**F**	**r**	**p**	**F**	**r**	**P**	**F**	**r**	**p**	**F**	**r**	**p**	**F**	**r**	**p**	**F**	**r**	**p**	**F**
Age		0.66			0.69			0.3			0.27			0.55			0.61			0.68	
BMI		0.16			0.37			0.28			0.58			0.86			0.17			0.1	
WC	-0.35	0.01	0.4	-0.33	0.01	0.58		0.13			0.64		-0.33	0.01	6.5	0.33	0.01	0	-0.27	0.05	1.83
SBP		0.62			0.72			0.75			0.81			0.37			0.21			0.56	
Duration of HD		0.56			0.59		0.31	0.02	14.43		0.06			0.25			0.76			0.19	
Smoking (current)		0.46			0.33			0.32			0.27			0.23			0.86			0.69	
Hb		0.72			0.98			0.47			0.23			0.63			0.53			0.83	
WBC	-0.38	0.01	7.53	-0.37	0.01	6.21		0.13			0.66		-0.31	0.03	0.64	0.44	0	9.99		0.19	
Cr		0.25			0.35			0.28			0.87			0.77			0.48			0.45	
BUN		0.07			0.09			0.18			0.51			0.44			0.15			0.4	
Alb		0.11			0.21			0.93			0.3			0.81			0.05			0.38	
adjusted-Ca		0.47			0.82			0.72			0.28			0.5		0.32	0.03	1.35		0.72	
IP	0.29	0.05	0		0.08		0.35	0.02	2.02		0.81			0.81			0.15			0.06	
K		0.41			0.19			0.16			0.59		0.31	0.04	4.59		0.28			0.31	
Mg		0.17			0.27			0.38			0.32			0.45			0.15			0.99	
UA		0.57			0.88			0.13		0.3	0.04	4.46		0.33			0.15			0.94	
Intact-PTH		0.22			0.31			0.15			0.1			0.84			0.6			0.95	
β2MG		0.24			0.43			0.18			0.33			0.49			0.36			0.56	
BS		0.68			0.88			0.91			0.17			0.69			0.68			0.33	
LDL-C		0.9			0.83			0.6			0.69			0.36			0.34			0.77	
TG	-0.4	0	0.04	-0.37	0.01	0.54		0.23			0.07		-0.3	0.03	1.36	0.52	<0.0001	2.37	-0.31	0.02	5.96
HDL-C	0.55	<0.0001	19.16	0.45	0	24.01		0.41			0.13			0.27		-0.61	<0.0001	25.35		0.12	
CRP		0.92			0.87			0.73			0.16			0.79			0.73			0.38	

Parameters with p<0.05 were subsequently entered into multiple regression analysis as significant and independent variables. Parameters with F>4.0 were significant and independent variables. Abbreviations as in Table 1.

The HMW-APN/Total-APN ratio correlated significantly and negatively with WC, WBC and TG, and positively with K. Stepwise regression analysis that included WC, WBC, K, and TG identified WC and K as significant and independent determinants of the HMW-APN/Total-APN ratio. The C1q-APN/Total-APN ratio correlated significantly and negatively with HDL-C, and positively with WC, WBC, adjusted-Ca and TG. Stepwise regression analysis that included WC, WBC, adjusted-Ca, TG, and HDL-C identified WBC and HDL-C as significant and independent determinants of the C1q-APN/Total-APN ratio. The C1q-APN/C1q ratio correlated significantly and negatively with WC and TG. Stepwise regression analysis that included WC and TG identified TG as the only significant and independent determinant of the C1q-APN/C1q ratio.

### Simple and multivariate logistic regression analyses of ACVD

Simple logistic regression analysis was used to evaluate the relationship between ACVD and various serum adiponectin parameters (Table 4). Sex, hypertension, smoking status (current-smoker), IP, C1q-APN, and C1q-APN/C1q correlated significantly with ACVD (Model 1; no adjustment). Multiple regression analysis identified IP and C1q-APN (Model 2) and C1q-APN/C1q ratio (Model 3) as significant determinants of ACVD in HD patients (Table 4).

**Table 4:** Correlations between clinical features of patients on maintenance hemodialysis and ACVD

	**Model 1: No adjustment**		**Model 2: Multivariate (C1q-APN)**	**Model 3: Multivariate (C1q-APN/C1q)**
	**r**	**p**	**p**	**p**
Age	0.05	0.12	-	-
Sex (Male)	0.22	0	-	-
BMI		0.25		
WC		0.3		
Duration of HD		0.53		
HT	0.12	0.04	0.53	0.51
DM		0.16		
DL		0.28		
Smoking (current)		0.04	0.53	0.53
Cr		0.63		
Adjusted-Ca		0.73		
IP	0.2	0.01	0.01	0.01
K		0.61		
Mg		0.59		
UA		0.29		
intact-PTH		0.53		
β2MG		0.14		
CRP		0.19		
Total-APN		0.13		
HMW-APN		0.29		
C1q-APN	-0.13	0.03	0.04	
C1q		0.43		
HMW-APN/Total-APN		0.92		
C1q-APN/Total-APN		0.54		
C1q-APN/C1q	-0.15	0.02		0.02

Parameters with p<0.05 in Models 2 and 3 were subsequently entered into multiple logistic regression analysis as significant and independent variables. Abbreviations as in Table 1. Model 2 (adopted factors; age, sex, HT, smoking, IP, C1q-APN). Model 3 (adopted factors; age, sex, HT, smoking, IP, C1q-APN/C1q).

## Discussion

The following were the major findings of the present study in HD patients: 1) serum C1q-APN, Total-APN and HMW-APN were lower in males than in females; however, serum C1q-APN/Total-APN ratio was not different between the two sexes, 2) stepwise regression analysis identified HDL-C as the only significant and independent determinant of C1q-APN in males, and duration of HD as the only significant and independent determinant of C1q-APN in females, 3) stepwise regression analysis identified UA, LDL-C and TG as significant and independent determinants of the C1q-APN/Total-APN ratio in males, and WBC and HDL-C as significant and independent determinants of the C1q-APN/Total-APN ratio in females, and 4) multiple regression analysis identified IP and the C1q-APN or C1q-APN/C1q ratio as significant determinants of ACVD in HD patients, whereas there was no relationship between the C1q-APN/Total-APN ratio and ACVD.

Disorders of mineral metabolism, such as hyperphosphatemia, hypercalcemia, and secondary hyperparathyroidism, are independently associated with mortality and morbidity of cardiovascular diseases in HD patients [11-14]. The present study also showed that high levels of serum IP correlated with ACVD (Table 4). These results suggest that diet therapy, such as low nitrogen and phosphorus intake, are probably important for prevention of ACVD in HD patients. Matsubara et al. reported that increased adiponectin may contribute to the suppressive bone marrow function [15]. The present study investigated the biochemical and haematological parameters and C1q-APN paremeters. Stepwise regression analysis identified WBC counts as a significant and independent determinant of C1q-APN/Total-APN ratio in females (Table 4) but not males (Table 3). More studies are required to confirm the findings and elucidate the biological mechanisms underlying the association between the haematological parameters and C1q-APN.

Smoking and adiponectin are individually associated with cardiometabolic pathologies. A systematic review reported that there is a decreased adiponectin level in current smokers and this reduction is reversed by quitting smoking [16]. In HD patients, smoking status (current-smoker) also correlated significantly with ACVD (no adjusted, Table 4), however there were no significant correlations between C1q-APN, C1q-APN/Total-APN, C1q-APN/C1q and smoking status in both male (Table 2) and female HD patients (Table 3). More studies are required to confirm the findings.

HD patients have much higher levels of adiponectin, compared with the general population [4]. However, low circulating levels of adiponectin independently predict cardiovascular and mortality outcomes in HD patients, the relationship being extensively confounded by various patient-related factors [17-22]. The cross-sectional preset study found that Total-APN did not associate with ACVD (Table 4). Complement activation and C1q binding activity have been described in HD patients [23]. Inoshita et al. [24] found significantly higher levels of functional complement activity of all three pathways, i.e., the classical pathway, the alternative pathway, and the lectin pathway, in HD patients than healthy controls. The present study showed higher serum C1q levels in HD male patients (59.7 ± 1.2 μg/mL, Table 1) than the men of the general population (56.0 ± 10.0 μg/mL) [5]. We recently reported that serum C1q-APN/Total-APN ratio correlated with the metabolic syndrome in men [5], and with polyvascular diseases and coronary artery disease in type 2 diabetics [7,8], although there was no significant difference in serum C1q-APN levels between patients without and with coronary artery disease. In HD patients, low serum C1q-APN, but not C1q-APN/Total-APN ratio, correlated with ACVD, independent of age-, sex-, other ACVD risk factors (hyperphosphatemia) (Table 4), a finding described for the first time to our knowledge. However, the different association of adiponectin-C1q with ACVD in between diabetic and HD patients remains unclear. Further studies are necessary to elucidate the pathophysiological role of C1q-APN in HD patients. Future monitoring of the long-term effects of serum C1q-APN and C1q on the cumulative incidence of cardiovascular events in HD patients is required.

The present study has several limitations. First, this is a cross-sectional study, making it difficult to establish a cause-effect relationship. Second, all patients in this study were Japanese and any differences from other ethnicities are unknown. Third, there is a potential bias in single center trials. Fourth, the number of patients was relatively small. The study included a limited number of patients and further studies of larger sample should be conducted in the future.

## Conclusions

The present study demonstrated for the first time that serum C1q-APN was lower in male HD patients than in females, and that lower serum C1q-APN and C1q-APN/C1q ratio, but not C1q-APN/Total-APN, correlated with ACVD in HD patients. These results suggest that the network of adiponectin and C1q may play a role in the pathophysiology of ACVD in HD.

## Abbreviations

ACVD: Atherosclerotic cardiovascular disease;Alb: Albumin;β2MG: β2-microglobulin;BMI: Body mass index;BUN: Blood urea nitrogen;BS: Blood glucose;C1q-APN: C1q-binding adiponectin;Cr: Creatinine;CRP: C-reactive protein;DBP: Diastolic blood pressure;DL: Dyslipidemia;DM: Diabetes mellitus;ELISA: Enzyme-linked immunosorbent assay;HD: Hemodialysis;HDL-C: High density lipoprotein-cholesterol;HMW: High-molecular weight;HMW-APN: High molecular weight-adiponectin;intact-PTH: intact parathyroid hormone;LDL-C: Low density lipoprotein-cholesterol;RBC: Red blood cell count;SBP: Systolic blood pressure;Total-APN: Total-adiponectin;UA: Uric acid;WBC: White blood cell count;WC: Waist circumference

## Competing interests

KK, TF and IS are promotional speakers for Otsuka Pharmaceutical Co., Ltd. TF is a member of the “Department of Metabolism and Atherosclerosis”, a sponsored course endowed by Kowa Co. Ltd. The company has a scientific officer who oversees the program. All other authors declare no competing interests. Human serum C1q-binding adiponectin complex assay is under patent application in Japan.

## Authors’ contributions

KK researched, collected, analyzed the data, participated in the concept and design of the study, interpretation of data and reviewed/edited the manuscript. NK and MA recruited the patients and collected the data. HN and HK analyzed the data.TF and IS contributed to the discussion and wrote the manuscript. All authors read and approved the final version of the manuscript.

## Pre-publication history

The pre-publication history for this paper can be accessed here:

http://www.biomedcentral.com/1471-2369/14/50/prepub
